# Okra polysaccharide mitigates carrageenan-induced thrombosis in mice by regulating inflammation and oxidative stress

**DOI:** 10.3389/fphar.2025.1576108

**Published:** 2025-04-10

**Authors:** Jinping Ni, Xiwen Cao, Xianqing Hu, Shenwen Fu, Meixiu Jiang, Yuqi Ni

**Affiliations:** ^1^ Department of Cardiology, Affiliated Jinhua Hospital, Zhejiang University School of Medicine, Jinhua, China; ^2^ School of Food Science and Technology, Jiangnan University, Wuxi, China; ^3^ The QUEEN MARY School, Jiangxi Medical College, Nanchang University, Nanchang, China; ^4^ Department of Central Laboratory, Affiliated Jinhua Hospital, Zhejiang University School of Medicine, Jinhua, China; ^5^ The National Engineering Research Center for Bioengineering Drugs and the Technologies, Institute of Translational Medicine, Jiangxi Medical College, Nanchang University, Nanchang, China

**Keywords:** carrageenan, thrombosis, OP, platelet, inflammation, oxidative stress

## Abstract

**Introduction:**

Thrombosis is a serious health hazard, which has been paid more and more attention.Okra polysaccharide (OP) is a biologically active substance extracted from okra which exhibits anti-inflammation and anti-oxidative properties. Nevertheless, the effect of OP on thrombosis is still unknown. In this study, we determined whether OP can suppress carrageenan-induced mice thrombosis and its involved mechanism.

**Methods:**

Twenty-four BALB/c mice were assigned to four groups randomly (6 mice/group): Ctrl, Model, OP low lose (OP-L, 200 mg/kg body weight), and OP high lose (OP-H,400 mg/kg body weight) were administered via intragastric administration for 9 days. Tails were photographed before collecting for H&E and Masson staining. Liver and lung tissues were collected for H&E staining, RT-qPCR, Western blot and GSH content detection. Injury or dysfunction of endothelial cells (ECs) was assessed using RT-qPCR, Western blot and cell adhesion assays.

**Results:**

OP can effectively improve carrageenan-induced thrombosis in tissues of mice (tail, liver, and lung) in vivo. In addition, OP inhibited inflammation by suppressing the toll-like receptor 4 (TLR4)/nuclear factor κB (NF-κB) pathway and reduced oxidative damage by elevating the level of GSH and antioxidant enzyme in liver and lung tissues. *In vitro*, OP inhibited thrombin-induced human platelet clots retraction, and decreased lipopolysaccharide (LPS)-activated adhesion of THP-1 monocytes to human umbilical vein endothelial cells(HUVECs) by suppressing intercellular adhesion molecule-1 (ICAM-1) level.

**Discussion:**

In conclusion, OP can inhibit thrombosis in mouse model by regulating inflammation and oxidative stress, which suggest that OP could act as a potential functional food for prevention of thrombus.

## Graphical Abstract

**Figure:**
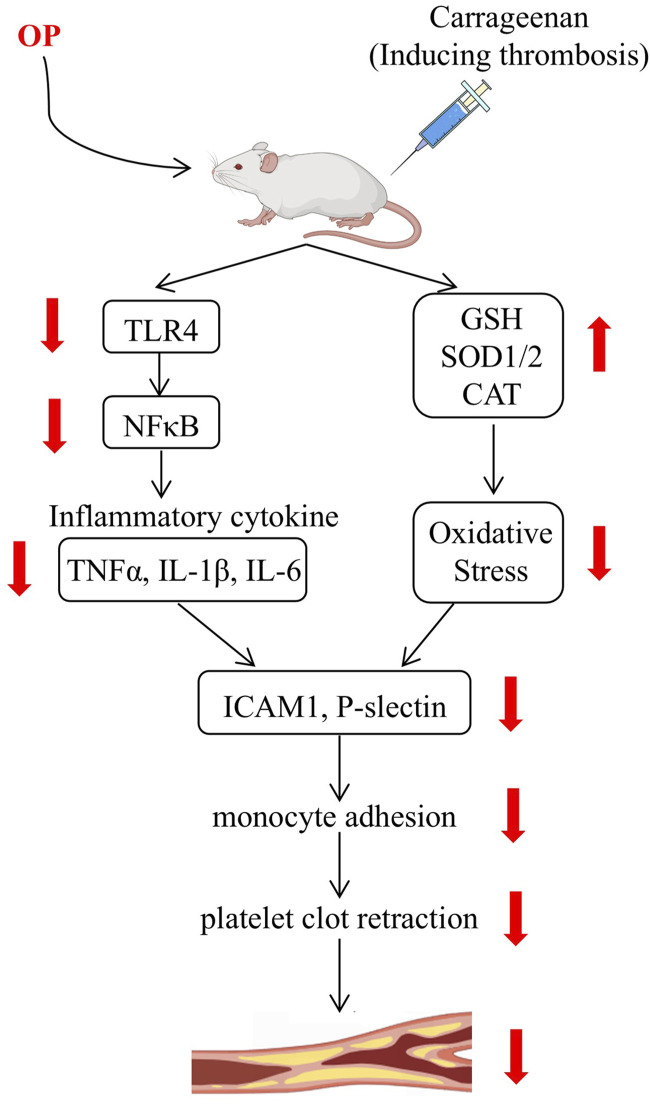

## 1 Introduction

Cardiovascular disease is closely related to morbidity and mortality in humans world-wide and is the leading cause of reduced quality of life (Roth et al., 2020). A thrombus is formed by blood flow on the surface of the inner surface in the blood vessel of the cardiovascular system at the spalling or repair site. It consists of insoluble fibrin, deposited platelets, accumulated white blood cells, and entrapped red blood cells, collectively causing thrombosis—a chronic, multifactorial condition marked by the formation of blood clots within arteries (Zhang et al., 2021; Lippi et al., 2011). It can occur in both arteries and veins across various tissues, and venous thrombosis potentially leads to chronic venous thromboembolism and pulmonary embolism (Koupenova et al., 2017; Li et al., 2019). Conversely, the arterial thrombosis may result in acute severe cardiovascular events, such as myocardial infarction and stroke (Caron and Anand, 2017; Lee et al., 2023). On the whole, thrombosis is a major threat to human health and has drawn growing attention.

Thrombus formation involves a complex series of steps, including the release of prethrombotic substances, platelet activation, aggregation, adhesion, and thrombine-mediated fibrin deposition (Lippi et al., 2011; Alkarithi et al., 2021). It begins with vascular injury which contributes to platelet activation and overactivated platelets produce soluble agonists, including adenosine diphosphate (ADP), thrombin, and thromboxane A2, which can further promote platelets adhere to subendothelial exposed collagen, thereby mediating excessive aggregation to form thrombi (But et al., 2023; Zhang et al., 2022). Moreover, damaged endothelial cells are further activated by the adhesion of activated platelets, fibrin, and other cells, thereby increasing the expression of vascular cell adhesion molecule 1 (VCAM-1), ICAM-1, P-selectin, and E-selectin which in turn enhance the adhesion/aggregation of platelets to endothelium, and further promote the progression of thrombus (Gkaliagkousi et al., 2007; Yau et al., 2015; Li et al., 2018).

More and more studies have proved that the process of inflammation and thrombosis is closely linked, and inflammatory conditions such as infection, chronic autoimmune diseases and potentially uncertain clonal hematopoiesis are associated with an increased risk of thrombotic events in the clinic, which provides sufficient evidence for the relationship between inflammation and thrombosis (Stark and Massberg, 2021). Although thrombosis and inflammation have different physiological processes, there is a strong interdependence between their mechanisms. Inflammation has the potential to amplify the process of thrombus formation within blood vessels. Research indicates that inflammatory cytokines such as tumor necrosis factor-α (TNF-α) and interleukin-1β (IL-1β) facilitate the recruitment of leukocytes and the activation of endothelial cells, which may lead to compromised endothelial cell integrity and apoptosis (But et al., 2023; Lester et al., 2012). In addition, the vascular endothelium is extensively damaged by oxidative stress caused by reactive oxygen species (ROS), which is an important factor in inducing and exacerbating thrombosis. Upon stimulation, the vascular endothelium produces a significant quantity of ROS, and excess ROS leads to increased protein oxidation, permeability of the endothelial barrier, adhesion molecules, and inflammation, which in turn accelerates intravascular oxidative stress, then results in promoting thrombus. Anyway, oxidative stress triggers thrombosis, and in turn, thrombosis aggravates oxidative stress (Wang et al., 2022; Wang et al., 2020; Mezzano et al., 2001).

Despite the notable advances in medicines to prevent or treat thrombosis, such as urokinase, aspirin, clopidogrel or ticagrelor, several drawbacks are still with these medicines. For instance, intravenous injections are required for urokinase. Some serious adverse effects, such as liver and/or kidney dysfunctions and gastrointestinal bleeding may be caused by aspirin or clopidogrel (Li et al., 2019; Kunamneni et al., 2007). Adverse effects of ticagrelor include dyspnea and bradycardia (Valette et al., 2018). Hence, it is essential to discover novel medicines which has effective antithrombotic properties and minimal side effects. Presently, a growing amount of research has indicated that plant-derived natural products may significantly alleviate the progression of thrombotic disorders due to the affordability, high safety, and multiple targets. Consequently, development of effective and safe natural therapies for treatment of thrombotic diseases is more in with the needs of patients.

Okra polysaccharide (OP) is a biologically active substance isolated from okra [*Abelmoschus esculentus* (L.) Moench], which is a nutritious and flavorful vegetable with outstanding biological activities and health benefits. Studies have confirmed that OP exhibits multiple pharmacological characteristics, including antioxidant, hypoglycemic, immunoregulatory, anticancer, and hepatoprotective actions (Yan et al., 2023; Zhu et al., 2020; He et al., 2024). OP has the potential to improve diabetic nephropathy in diabetic mice via stimulating AMPK-Sirt1-PGC-1α signaling pathway to inhibit oxidative stress and apoptosis (Liao et al., 2019a). Additionally, it exerts anti-T2DM properties partially by regulating oxidative stress through PI3K/AKT/GSK3β pathway-medicated Nrf2 transport (Liao et al., 2019b). Fan et al. (2013) reported that the dietary supplement of OP could reduce total serum cholesterol levels, blood glucose, and body weight in high-fat diet-fed C57BL/6 mice. Another study revealed that OP could enhance B-lymphocytes propagation and spleen weight by inhibiting the NF-кB transcription factor in mice with bacterial infections (Wahyuningsih et al., 2018). Recently, many studies have highlighted the neuroprotective properties of OP, which can mitigate cognitive injury associated with metabolic syndrome (Yan et al., 2020a; Hsiao et al., 2024), and can exert antidepressant effects through anti-inflammation and rebalancing the gut microbiota (Yan et al., 2020b). Furthermore, OP treatment demonstrated anti-colorectal cancer (CRC) effects in a mouse model exposed to azoxymethane (AOM) and dextran sodium sulfate (DSS) (Deng et al., 2023). It was also noted that OP has a dual role of immunomodulation against carcinogenic liver injury in mice (Hayaza et al., 2021).

Although OP has proven to be of great practicality and has a wide range of medical applications, its role in the formation of thrombosis is still blank, and the mechanisms on regulating thrombosis formation remain unclear. Based on the anti-inflammatory and antioxidant function of OP and the safe natural molecular structure, we speculate that OP has a promising role in the treatment of thrombosis. In this work, we developed a thrombus model in mice by carrageenan, and fed mice with OP for determination the protective role of OP on thrombosis and to explore the involved mechanisms.

## 2 Materials and methods

### 2.1 Reagents

OP were bought from ZhanXun Biotechnology Co., Ltd. (Xian, China), and the polysaccharide content is over 95%. κ-Carrageenan, LPS and aspirin were obtained from Sigma-Aldrich (St. Louis, MO, USA). Rabbit anti-SOD1 (E4G1H, Cat# 37385S), SOD2 (D9V9C, Cat# 13194S), MMP2 (Cat# 87809S), NF-κB P65 (Cat# 8242) and NF-κB pp65(Cat# 3033) monoclonal antibodies were acquired from Cell Signaling Technology (Danvers, MA, USA). Mouse anti-VCAM-1 (E-10, Cat# sc-13160) monoclonal antibody was obtained from Santa Cruz Biotechnology (Santa Cruz, CA, USA). The rabbit anti-TLR4 polyclonal antibody (Cat# ab13556) was acquired from Abcam (Cambridge, MA, USA). Rabbit anti-MMP9 (Cat# 10375-2-AP) polyclonal antibody and Mouse anti-GAPDH (Cat# 60004-1-Ig) monoclonal antibody were acquired from Proteintech Group (Chicago, IL, USA).

### 2.2 Animals

All animal study protocols were performed in accordance with the NIH’s Guide for the Care and Use of Laboratory Animals, and received approval from the Experimental Animal Welfare and Ethics Committee at Affiliated Jinhua Hospital, Zhejiang University School of Medicine (Approval No. AL-JHYY202356).

Male BALB/c mice, aged 7 weeks and weighing 25 ± 1 g, were obtained from Ziyuan Laboratory Animal Technology Co. Ltd. (Hangzhou, China). The mice were kept in controlled environment with a temperature maintained at 23°C ± 3°C and a light cycle consisting of repeated 12-h dark/12-h light. They were given about 1 week to adjust to the laboratory conditions prior to the initiation of experiments.

### 2.3 Carrageenan-induced thrombosis mouse model

Carrageenan, a type of mucopolysaccharide obtained from red algae, has the potential to induce intense inflammation, resulting in damage to vascular ECs and subsequently leading to the formation of thrombosis (Wang et al., 2012). The formation of thrombosis in mice induced by carrageenan, especially within the tail vessels, serves as a suitable animal model for assessing the antithrombotic properties of a medicament.

**FIGURE 1:**
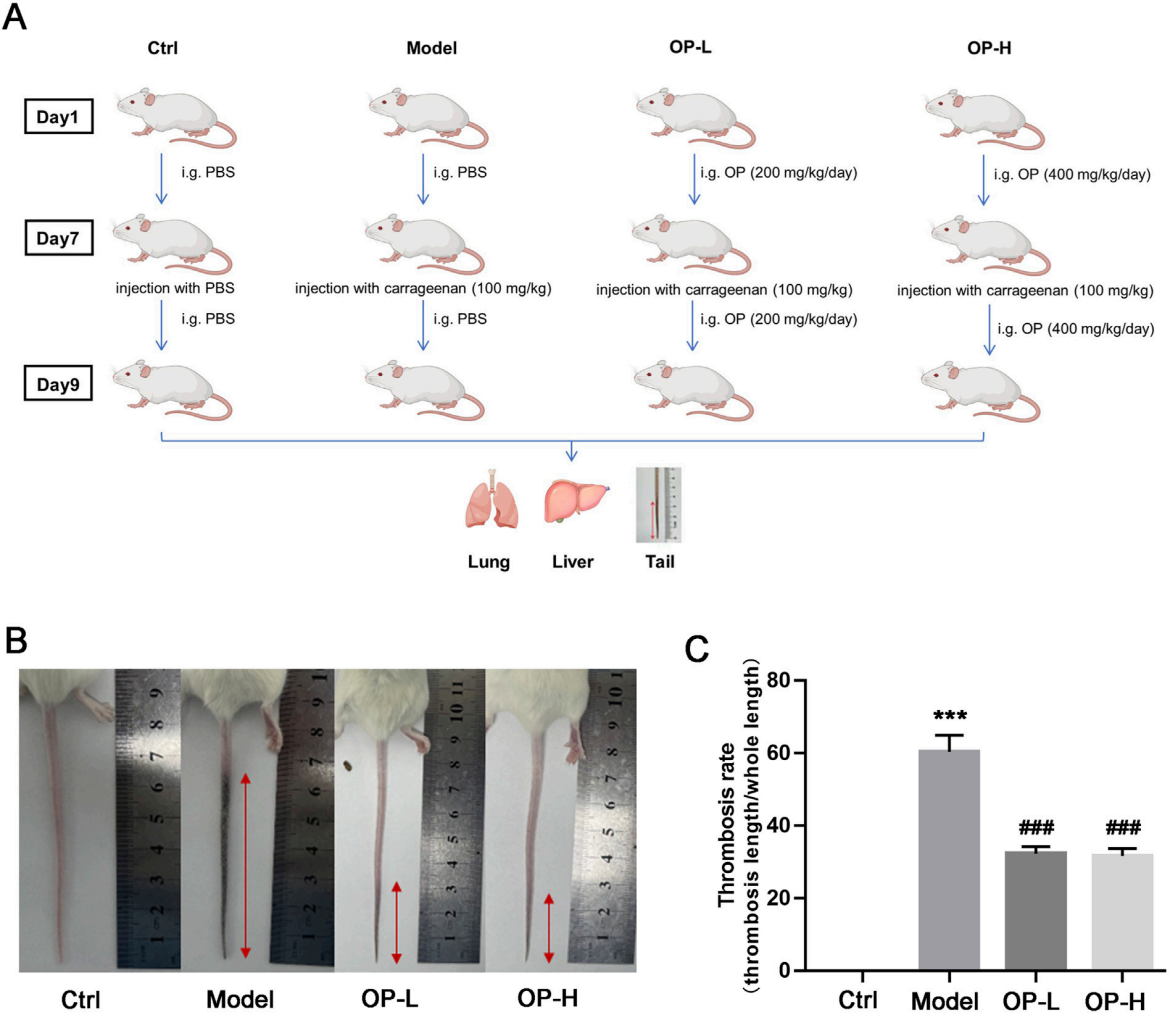
OP inhibits carrageenan-induced thrombosis in mouse tail. **(A)** Design experimentation: BALB/c mice were divided into four groups (*n* = 6) and were subjected to the treatments described in methods. **(B)** The tail of each mouse was captured in photographs, and the images that are representative are displayed. **(C)** The rate of thrombosis was assessed by measuring the proportion of the tail length impacted by thrombus in relation to the overall tail length. ***p < 0.001 vs Ctrl; ^###^ *p* < 0.001 vs Model (*n* = 6).

To assess the impact of OP on thrombosis caused by carrageenan in the blood vessels of the mouse tail, as well as in liver and lung tissues, a total of twenty-four BALB/c mice were assigned to four groups randomly (6 mice/group): Ctrl, Model, OP low lose (OP-L, 200 mg/kg body weight), and OP high lose (OP-H, 400 mg/kg body weight). Mice were subjected to treatment as illustrated in Figure 1A. In brief, the mice in Ctrl, Model or treatment (OP) group received intragastric administration of PBS or OP for a duration of 9 days. On day 7, 100 mg/kg body weight carrageenan was i.p injected into each mouse with the exception of the control group, which received PBS injections. Following the experiment, mice were anesthetized using a CO_2_ chamber, and their tails were photographed before collecting the samples of mouse tail, liver, and lung.

### 2.4 Hematoxylin and eosin (H&E) and masson staining

A section of the mouse tail, taken from designated points (measured from the tip of the tail), along with samples of liver or lung tissue, were collected and fixed in 4% paraformaldehyde for a duration of 24 h. Following dehydration, these tissue samples were embedded in paraffin and subsequently cut into sections of 5 μm thickness. To evaluate the presence of thrombi in mouse tissue vasculature, sections were subjected to H&E staining according to the general procedure. For evaluating the content of collagen, the mouse tail slices were performed Masson staining using a specific assay kit (Solarbio, Beijing, China). Images from both Masson and H&E stains were captured using a Leica microscope (Wetzlar, Germany).

### 2.5 Cell culture

HUVECs (Human umbilical vein endothelial cells) and THP-1 monocytes (a human monocytic cell line) were acquired from ATCC (Manassas, VA, USA). HUVECs were grown in endothelial cell medium (ECM) sourced from ScienCell (#1001), supplemented with containing 1x endothelial cell growth supplement (ECGS), penicillin/streptomycin, and 5% fetal bovine serum. Meanwhile, culture of THP-1 monocytes was established in 1640 RPMI medium with 10% fetal bovine serum, 50 μg/mL penicillin, and 50 μg/mL streptomycin. When the cells reached approximately 90% confluence, they were transitioned to a serum-free medium prior to treatment. The concentrations of OP used for the *in vitro* experiments were established based on results from earlier studies (Xiong et al., 2021).

### 2.6 Assessment of monocyte adhesion to HUVECs

HUVECs grown in 24-well plates were exposed to either 200 ng/mL LPS or a combination of LPS and OP for a duration of 12 h. Subsequently, CFSE-labeled THP-1 monocytes (1 × 10^5^ cells/well) at a concentration of 5 μM were introduced to the HUVECs and incubated at 37°C for 30 min. Following three rounds of PBS washing to exclude non-adherent cells, the attached monocytes on HUVECs were examined under a Leica microscope (Wetzlar, Germany) and captured in photographs.

### 2.7 Isolation of platelets

The research protocol concerning human platelets was designed in line with the World Medical Association’s Code of Ethics and received approval from the Medical Ethics Committee of Jinhua Hospital, which is affiliated with Zhejiang University School of Medicine. Prior to collecting plasma from a healthy donor, written informed consent was secured from the individual(s) involved. Platelet isolation was carried out in accordance with earlier instructions (Li et al., 2019; Huang et al., 2024). In short, human plasma was centrifuged at 600×*g* for 10 min at ambient temperature to obtained platelets. The platelets were then washed and resuspended in a buffer solution containing 10 mM HEPES (pH 7.4), 140 mM NaCl, 3 mM KCl, 0.5 mM MgCl_2_, 5 mM NaHCO_3_, and 10 mM glucose for further applications.

### 2.8 Clot retraction assay

The platelet clot retraction assay was conducted following previously established methods (Zhang et al., 2022). In brief, human platelets (3 × 1 08/mL) were resuspended in a buffer solution composed of 10 mM HEPES (pH 7.4), 140 mM NaCl, 3 mM KCl, 0.5 mM MgCl_2_, 5 mM NaHCO_3_, and 10 mM glucose at an incubation temperature of 37°C. CaCl_2_ was added to achieve a final concentration of 1 mM, followed by treatment with PBS or OP (0.5 and 1 mg/mL) for an additional 30 min at the same temperature. After this incubation period, fibrinogen (2 mg/mL) was introduced to the mixture and thoroughly mixed before adding thrombin (1 U/mL) to initiate clot retraction. Photographs of the clots were taken at various time points during the process.

### 2.9 Determination of mRNA levels by quantitative real-time PCR (qRT-PCR)

Following treatment, total RNA was isolated as described previously (An et al., 2020). In brief, HUVECs or mouse tissues were lysed in Trizol. The lysate was mixed well with chloroform and centrifuged for 10 min using a Microfuge at 16,200 × *g* at 4°C. The top aqueous phase was collected and mixed with an equal volume of isopropanol to precipitatetotal cellular RNA. The cDNA was synthesized with 1 μg total RNA using an RT kit purchased from Vazyme and the following conditions were used: Initial wiper gDNA at 42°C for 2 min, followed by 50°C for 15 min and 85°C for 5 s. This was followed by qRT-PCR as previously reported (Zeng et al., 2023), utilizing the SYBR green PCR master mix provided by Vazyme, and the PCR-specific amplification was conducted with the Applied Biosystems VIIATM real-time PCR machine with the primers described in Table 1. The quantification of target mRNA was performed by the 2^−ΔΔCt^ method and GAPDH levels in the corresponding sample were used for normalization.

**TABLE 1:** Sequences of primers for qRT-PCR

Gene	Forward (5’---3’)	Reverse (5’---3’)
GAPDH	ACCCAGAAGACTGTGGATGG	ACACATTGGGGGTAGGAACA
TNFα	ATGGCCTCCCTCTCATCAGT	TTTGCTACGACGTGGGCTAC
IL-1β	AACCTGCTGGTGTGTGACGTTC	CAGCACGAGGCTTTTTTGTTGT
IL-6	GGGACTGATGCTGGTGACAA	TCCACGATTTCCCAGAGAACA
MMP2	TGGCAAGGTGTGGTGTGCGAC	TCGGGGCCATCAGAGCTCCAG
MMP9	GGTGTGCCCTGGAACTCACACG	AGGGCACTGCAGGAGGTCGT
P-selectin	GCATACTCATGGAATAACTCACG	GACGTCATTGAGGTGAGCG
SOD1	GCCTTGTGTATTGTCCCCAT	ACCATCCACTTCGAGCAGAA
SOD2	AGACACGGCTGTCAGCTTCT	CTGGACAAACCTGAGCCCTA
CAT	TATCTCCTATTGGGTTCCCG	CCGCAATCCTACACCATGTC

### 2.10 Evaluation of protein levels using western blot analysis

After treatment, total protein samples were extracted from HUVECs or mouse tissues using a protein lysis buffer (RIPA; cat. No. R0010; Beijing Solarbio Science & Technology Co., Ltd.) containing PMSF and cocktail (10 μg/mL), before being centrifuged at 10,000 × *g* for 10 min at 4°C (Jiang et al., 2017; Yu et al., 2016). After detection of protein content using a BCA assay kit (cat. no. 23227; Thermo Fisher Scientific, Inc.), the levels of TLR4, MMP2, MMP9, NF-κB, CAT, SOD1, SOD2 and GAPDH were assessed through Western blotting as previously outlined (Zeng et al., 2023; Wei et al., 2023). Briefly, proteins from each sample were separated by SDS/PAGE (10% gel) and transferred onto a nylon-enhanced nitrocellulose membrane. The membrane was then blocked with a solution of 5% skimmed milk in PBS for 1 h at room temperature, before being incubated with primary antibodies against TLR4 (1:1,000), MMP2 (1:1,000), MMP9 (1:1,000), NF-κB p65 (1:1,000), NF-κB p-p65 (1:500), CAT (1:1,000), SOD1 (1:1,000), SOD2 (1:1,000) and GAPDH (1:5,000) at 4°C overnight. This was followed by washing three times for 10 min each with a solution of 0.5% Tween-20 in PBS (PBS-T). The membranes were then exposed to HRP-conjugated goat anti-rabbit (1:5,000; cat. no. 31460; Thermo Fisher Scientific, Inc.) or anti-mouse (1:5,000; cat. no. 31430; Thermo Fisher Scientific, Inc.) IgG secondary antibodies and incubated for 1 h at room temperature. After three washes with PBS-T (10 min each), the membrane was incubated for 5 min in a mixture of equal volumes of Western blot chemiluminescence reagents 1 and 2 of the Ultra High Sensitivity ECL Kit (cat. no. GK10008; GlpBio Technology). The protein bands were visualized using an ECL detection system (Tanon Science and Technology Co., Ltd.) and analyzed using the ImageJ software (version 1.46r; National Institutes of Health).

### 2.11 Measurement of GSH content in liver and lung tissues

Briefly, liver or lung tissues were homogenized in iced-cold PBS (weight/volume = 1: 10). Then, the homog enized tissues were centrifuged at 10,000 × *g* (4°C) for 15 min. The supernatants were collected and used for GSH content evaluation by a commercial detection kit (Nanjing Jiancheng Institute of Biotech nology, Nanjing, China) according to the manufacturer’s instructions.

### 2.12 Data analysis

All data are presented as mean ± SEM, with representative results displayed. The statistical analyses were performed using version 7.0 of GraphPad Prism software. *p* values were determined via one-way ANOVA followed by Tukey’s test for multiple comparisons among more than two groups. *p* values for multiple comparisons of more than two groups in a one-way ANOVA were obtained using Tukey’s test. A *p* value of less than 0.05 was deemed statistically significant.

## 3 Results

### 3.1 OP mitigates thrombosis induced by carrageenan in mouse tails

To evaluate the effects of OP on both prevention and treatment of thrombosis, BALB/c mice were subjected to a regimen as shown in Figure 1A. Following 2 days post-carrageenan injection, images of the mouse tails were captured. As illustrated in Figure 1B, there was a notable formation of thrombosis in the Model group when compared to the Control group; however, mice that received OP treatment showed a significantly reduced length of tail affected by thrombosis compared to those in the Model group. Additionally, OP treatment led to a marked reduction in the increase of thrombosis rate caused by carrageenan (Figure 1C, *p* < 0.001).

**FIGURE 2:**
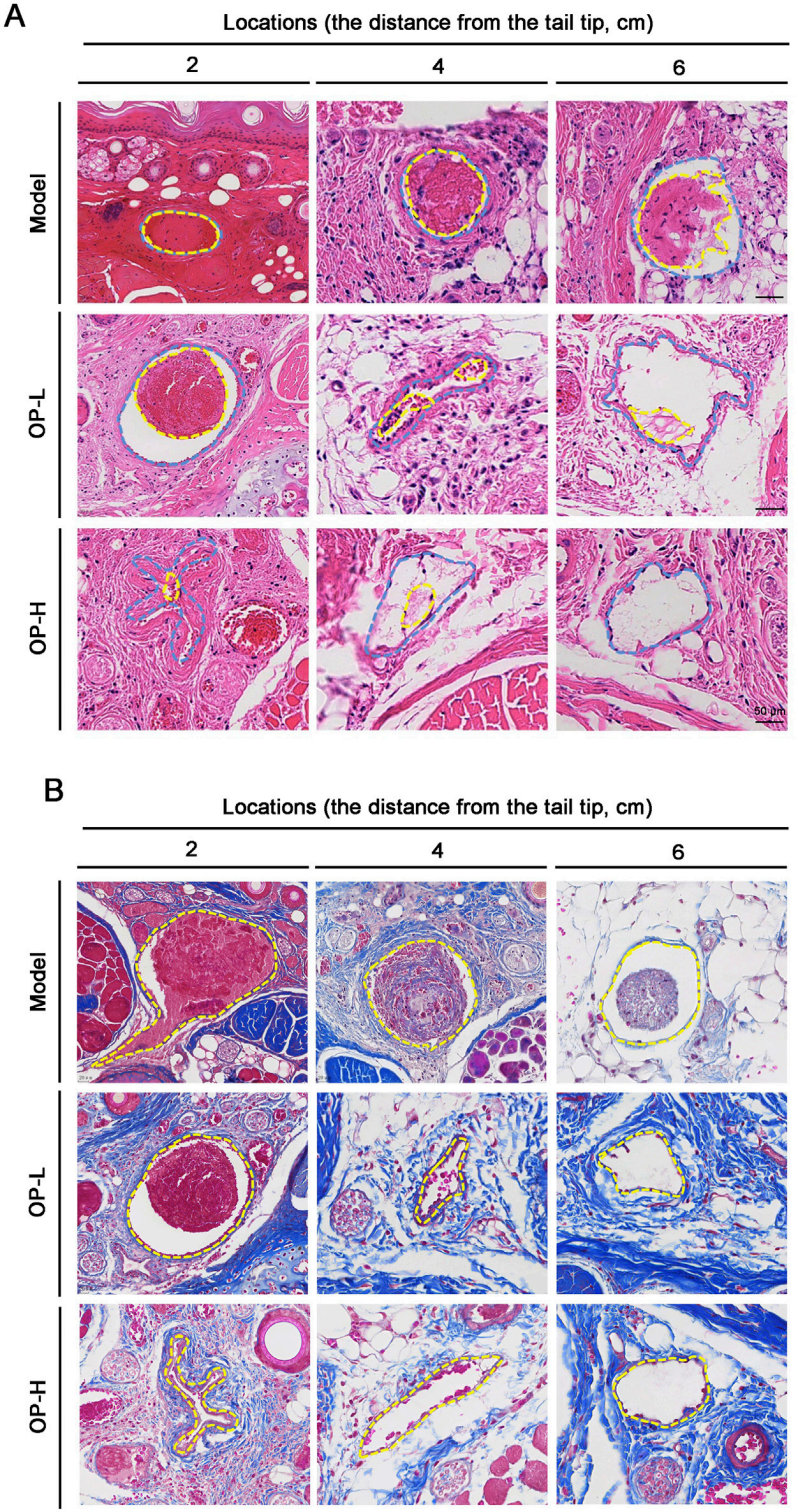
Pathological observation of tail tissues in mice with OP treatment. **(A)** H&E staining was conducted with the cross sections of the tail at several tail regions. The intravenous thrombus is shown by the yellow dotted line, while the blood vessel’s shape is shown by the blue dotted line. **(B)** Masson staining was conducted with the cross sections of the tail at several tail regions. The blood vessel’s outline is seen by the yellow dotted line.

To further validate the antithrombotic properties of OP, we conducted H&E staining with cross-sections of mouse tails at designated points. The results showed that in Model mice, tail vessels located 2 and 4 cm from the tip were entirely obstructed by thrombi. Conversely, OP led to a significant reduction in thrombosis within the tail vessels of treated mice, with little thrombus formation at distances of 4 and 6 cm (Figure 2A). Collectively, these findings unequivocally demonstrate that OP exerts preventive properties against thrombosis in mice.

Usually, the collagens located in the matrix beneath the vascular endothelial layer are not directly exposed to blood circulation. However, when endothelial cells are damaged, collagen is exposed which stimulates platelet activation and coagulation. That is to say, the contents of collagen within thrombi can indicate thrombus mass (Huang et al., 2024; Henke et al., 2006a). Consequently, we evaluated the collagen levels in thrombi by employing Masson staining on tail cross-sections taken at distances of 2, 4, and 6 cm. In contrast to the Model group, OP treatment resulted in a decrease in areas containing intrathrombotic collagen (Figure 2B).

### 3.2 OP suppresses thrombosis induced by carrageenan in mouse tissues

**FIGURE 3:**
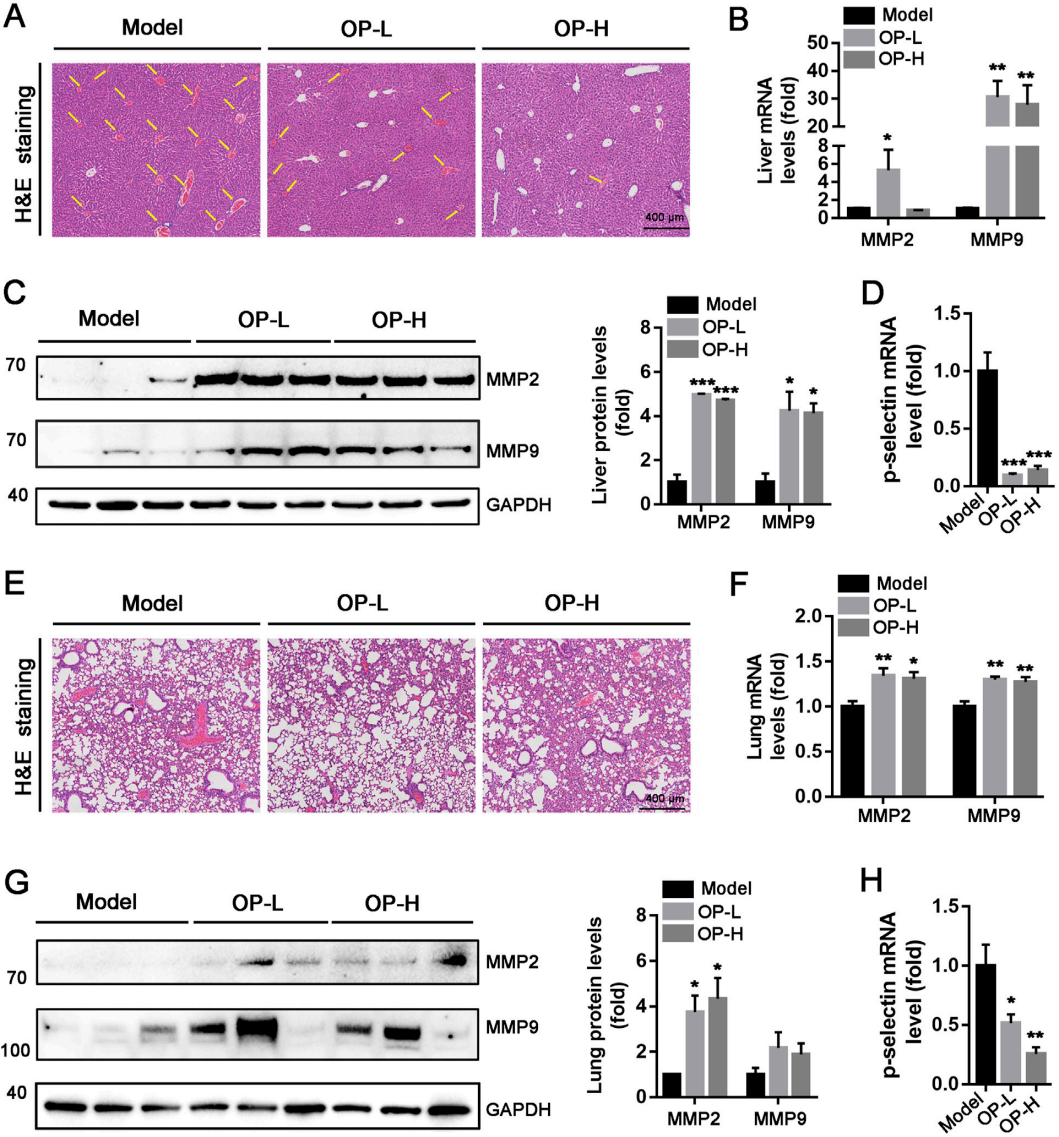
OP inhibits carrageenan-induced thrombosis in mouse liver and lung tissues. **(A, E)** H&E staining was conducted with the liver **(A)** and lung **(E)** cross sections. The intrahepatic thrombus development of mice is indicated by the yellow arrow. **(B–D, F–H)** Western blot and qRT-PCR were used to determine the level of MMP-2, MMP-9, and p-selection protein or mRNA in mouse liver **(B–D)** and lung **(E–F)**. *p < 0.05, **p < 0.01, ***p < 0.001 vs Model (*n* = 6).

Administration of carrageenan can lead to the development of tail thrombosis as well as vascular thrombosis in various other tissues. In line with the findings from mouse tails, a significant number of thrombi were found in the liver and lung vessels within the Model group, whereas only a few thrombi were identified in mice treated with OP (Figures 3A,E). Matrix metalloproteinase-2 (MMP-2) and MMP-9 are two enzymes involved in the degradation of collagen as thrombus resolution occurs (Li et al., 2018; Huang et al., 2024; Henke et al., 2006b). Consequently, we evaluated the effects of OP treatment on MMP-2 and MMP-9 expression levels in liver and lung tissues. The findings indicated that OP notably increased the expression levels of mRNA and protein for MMP-2 and MMP-9 in both mouse liver (Figures 3B,C, *p* < 0.05, *p* < 0.01, *p* < 0.001) and lung tissues (Figures 3F,G, *p* < 0.05, *p* < 0.01). P-selectin is identified as a thrombosis biomarker (Huang et al., 2024; Cambien and Wagner, 2004). Our research demonstrated that treatment with OP resulted in reduced levels of P-selectin mRNA in liver and lung tissues (Figures 3D,H, *p* < 0.001, *p* < 0.05). In summary, these findings indicate that OP has the potential to safeguard mice from carrageenan-induced thrombosis.

### 3.3 OP inhibits LPS-induced adhesion of THP-1 cells to HUVECs

**FIGURE 4:**
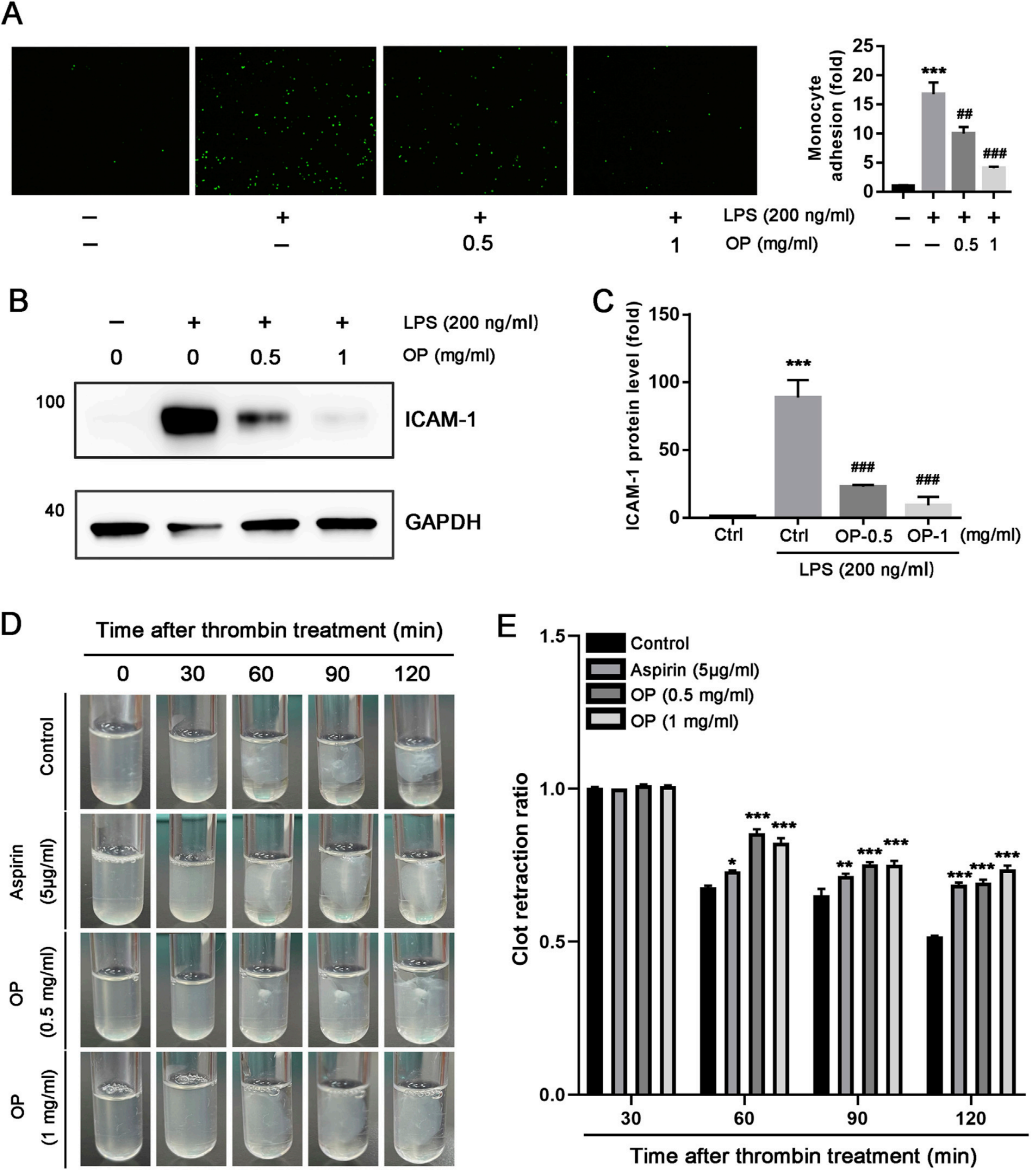
OP inhibits the adhesion of monocytes with HUVECs and thrombin-induced platelet activation. **(A)** Monocytes adhesion to HUVECs assay was performed with the labeled THP-1 monocytes and the treated HUVECs with LPS or OP. The count of adherent THP-1 cell in each group was measured and standardized to the count in control group. **(B, C)** Western blot was used to assess the expression of ICAM-1 protein **(B)** with quantitation of band density **(C)** in HUVECs treated with LPS and OP. **(D)** Clot retraction assay was conducted according to the description in methods, and photographs of the clots were taken at various time intervals. **(E)** Photoshop CS6 software was used to assess the area of the platelet clot spread. *p < 0.05,**p < 0.01,***p < 0.001 vs ctrl; ^##^ *p* < 0.01, ^###^ *p* < 0.001 vs LPS (*n* = 3).

The initial stage of thrombotic complications involves the attachment of monocytes to ECs. Consequently, our team conducted cell adhesion assays to examine the effect of OP on monocytes adhesion to ECs. As illustrated in Figure 4A, LPS significantly enhanced the THP-1 monocytes adhesion to HUVECs (*p* < 0.001). Nevertheless, treatment with OP significantly reduced the adhesion of THP-1 monocytes induced by LPS to HUVECs (*p* < 0.01, *p* < 0.001). Furthermore, we discovered that the expression of adhesion molecule ICAM-1 induced by LPS in HUVECs was substantially decreased by OP (Figures 4B,C, *p* < 0.001). In summary, the findings indicate that OP reduces the monocyte adhesion to HUVECs by downregulating ICAM-1 expression.

### 3.4 OP inhibits the retraction of platelets clots induced by thrombin *in vitro*

*In vitro*, thrombin can initiate platelet clot retraction which is a crucial step of thrombus consolidation (Lippi et al., 2011). To investigate whether OP has the ability to inhibit platelet aggregation, we isolated human platelets and applied thrombin to induce clot retraction. As illustrated in Figures 4D,E, thrombin effectively triggered the reaction of human platelet clot response, Both OP(0.5, 1 mg/mL) and aspirin (5 μg/mL) significantly inhibited platelet aggregation, and the inhibitory effect of OP(1 mg/mL) on platelet aggregation was better than aspirin (*p* < 0.001). Overall, these findings indicate that OP reduces thrombin-induced platelet clot retraction *in vitro*.

### 3.5 OP alleviates inflammation and oxidative stress in both liver and lung tissues

**FIGURE 5:**
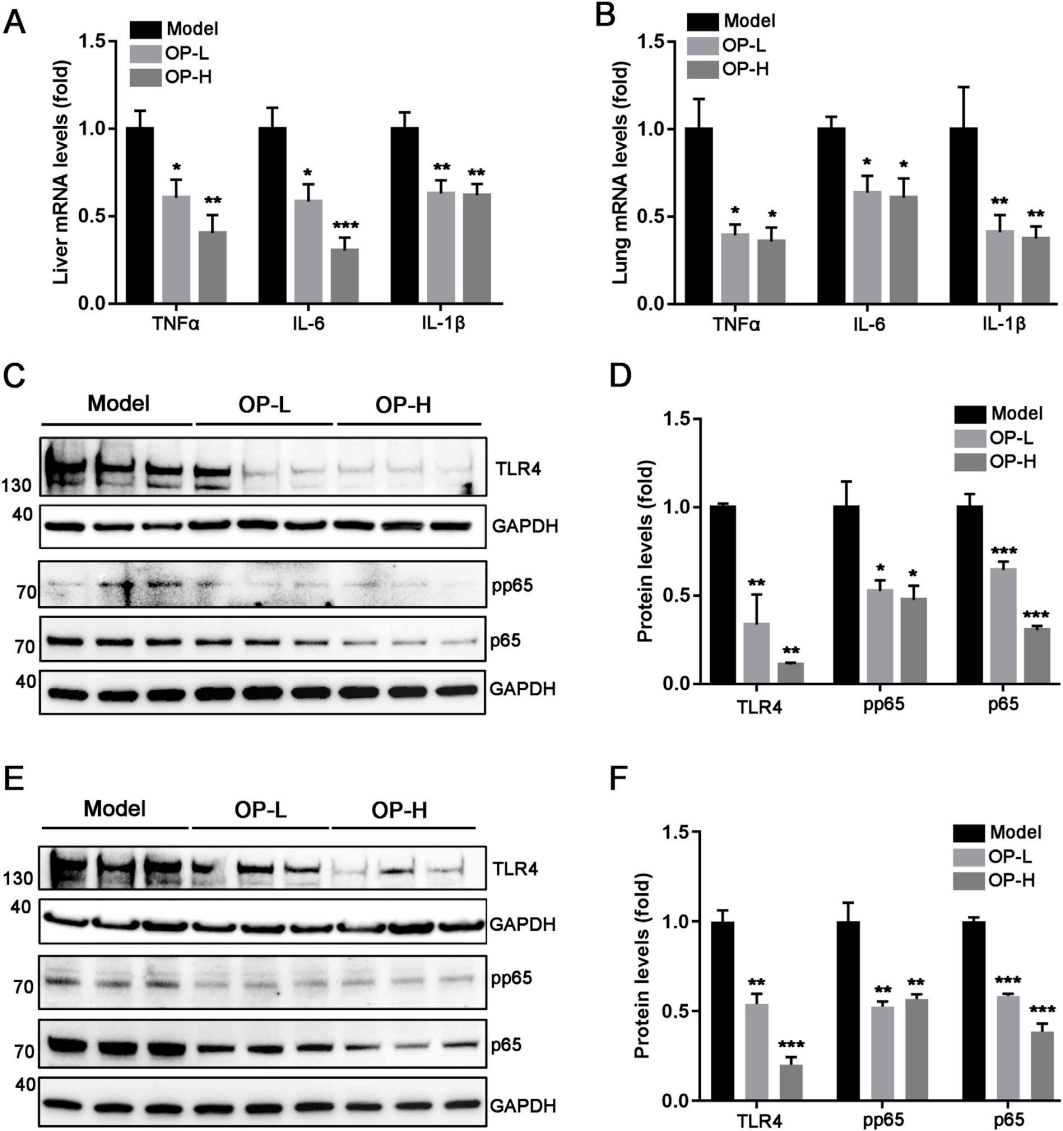
OP suppresses inflammation induced by carrageenan in mouse liver and lung tissues. **(A, B)** qRT-PCR was used to measure the level of TNF-α, IL-6, and IL-1β mRNA in the mouse liver **(A)** and lung **(B)**. **(C–F)** Western blot was used to examine the levels of TLR4, pp65, p65 and GAPDH in the liver **(C, D)** and lung **(E, F)** of mice, accompanied by quantification of band density. *p < 0.05, **p < 0.01, ***p < 0.001 vs Model (*n* = 6).

**FIGURE 6:**
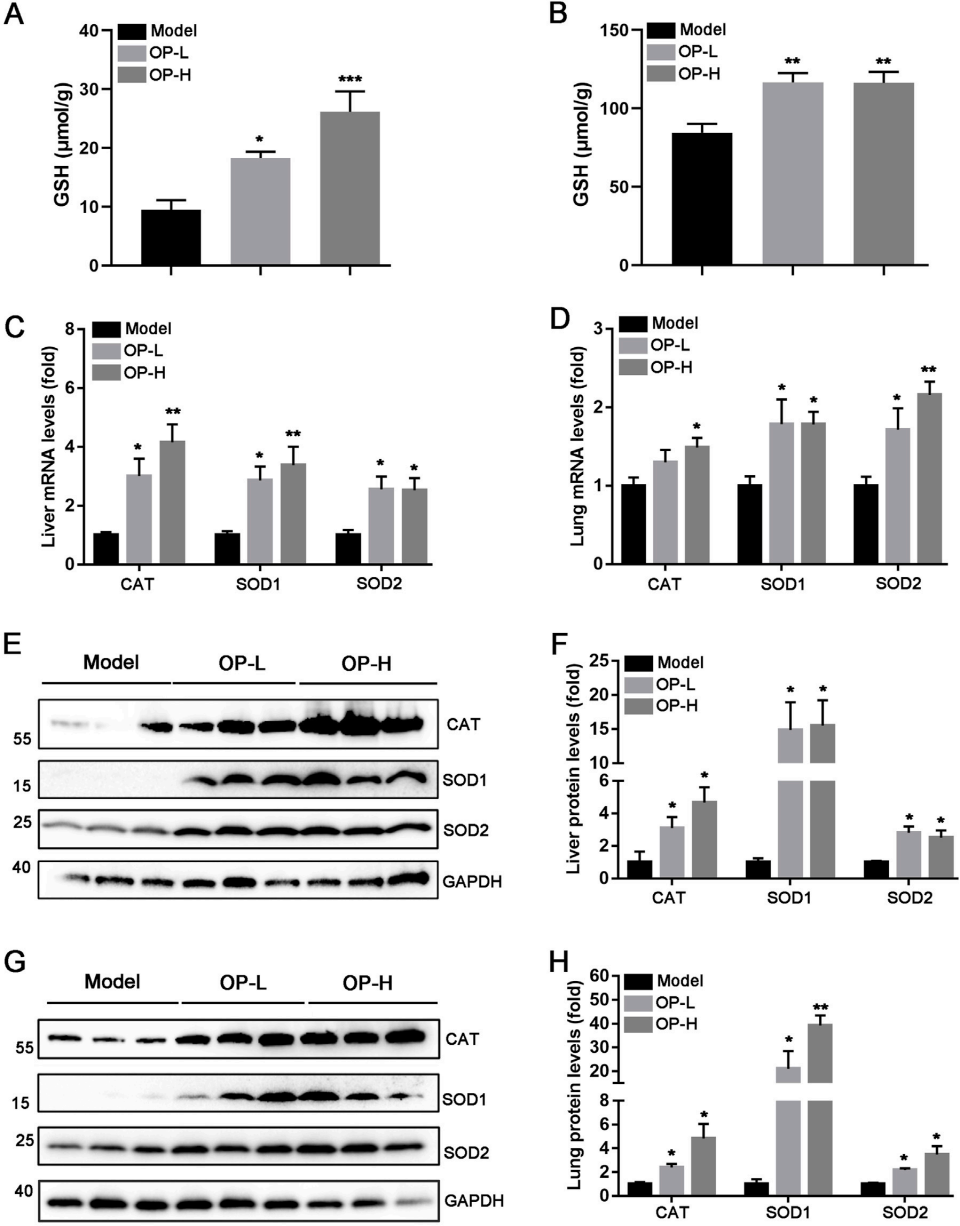
OP alleviates oxidative stress caused by carrageenan in the liver and lung tissues of mice. **(A, B)** The content of GSH in liver **(A)** and lung **(B)** tissues of mice was determined according to the kit. **(C, D)** qRT-PCR was used to determine the mRNA levels of CAT, SOD1, and SOD2 in the liver **(C)** and lung **(D)** tissues of mice. **(E–H)** Western blot was used to examine the levels of CAT, SOD1, SOD2, and GAPDH in the liver **(E, F)** and lung **(G, H)** tissues of mice, accompanied by quantification of band density. *p < 0.05, **p < 0.01 vs Model (*n* = 6).

The aforementioned findings indicate that OP diminished mice thrombosis induced by carrageenan. The progression of thrombosis is significantly promoted by oxidative stress and inflammation. Consequently, we hypothesized that OP might offer protection against liver and lung thrombosis triggered by carrageenan due to its capacity to mitigate inflammation and oxidative stress. The findings indicated that OP reduced mRNA levels of pro-inflammatory cytokines, like TNF-α, IL-1β, and IL-6, in the liver and lung tissues of mice (Figures 5A,B, *p* <0.05, *p* < 0.01, *p* < 0.001). Additionally, we observed that OP substantially decreased the protein levels of NF-κB and TLR4, as well as the phosphorylation of NF-κB in both liver and lung tissues (Figures 5C–F, *p* < 0.05, *p* < 0.01, *p* < 0.001). Furthermore, we determined the effects of OP on oxidative stress in liver and lung tissues. GSH (glutathione) is an important intracellular thiol antioxidant. Therefore, we examined the content change of GSH. Our findings indicated that OP significantly increased the content of GSH in both liver and lung tissues (Figures 6A,B, *p* <0.05, *p* < 0.01). Moreover, we found that OP not only elevated the mRNA levels of antioxidant enzymes such as SOD1, SOD2 and CAT (Figures 6C,D, *p* < 0.05, *p* < 0.01), but also increased their protein levels in both liver and lung tissues (Figures 6E–G, *p* < 0.05, *p* < 0.01). These findings indicate that the inhibition of thrombosis by OP may be due to its ability to reduce inflammation and enhance antioxidant levels.

## 4 Discussion

Thrombosis is a serious health hazard, which has been paid more and more attention. Despite the considerable progress made in preventing and treating thrombosis, certain medications still have associated side effects. Thus, it is crucial to identify new drugs that offer effective antithrombotic benefits while reducing potential adverse effects. In this research, we observed that OP reduces thrombosis caused by carrageenan in mice and improves thrombin-induced platelet clot retraction *in vitro*. Mechanistically, OP suppresses the TLR4/NF-κB pathway, resulting in decreased levels of inflammatory cytokines, while also boosts the ability to combat oxidative stress by promoting the expression of antioxidant enzymes. Our findings fill a gap in the field of OP in improving blood clots. The mechanism of OP in antithrombotic process was elucidated for the first time, and a new method of applying OP to the treatment of thrombosis was proposed.

The introduction of carrageenan leads to the formation of thrombi in veins, arterioles, and capillaries due to inflammation within the blood vessels of mouse tails. In severe cases, this can result in ischemic necrosis of the tail, which is visibly marked by a blackened appearance (Lee et al., 2023; Krill oil, 2010). Consequently, the degree of discoloration in the mouse tail acts as an important experimental marker for intuitively evaluating the severity of thrombosis. In our study, we found that OP reduced the thrombosis formation and thrombosis rate caused by carrageenan in mouse tails. Additionally, the injection of carrageenan can lead to thrombosis not just in the tail but also within blood vessels of various other tissues (Figures 1, 2). In line with the findings observed in mouse tails, we discovered that OP also diminished carrageenan-induced thrombosis formation in both liver and lung tissues (Figure 3). The findings mentioned above indicate that OP can restrain thrombosis induced by carrageenan in mice.

It is well recognized that inflammation plays a significant role in the formation of thrombosis. Inflammation within blood vessels can lead to thrombosis, while on the other hand, thrombi present in these vessels can aggravate inflammation (Poredos and Jezovnik, 2007). Thromboinflammation not only contributes to the thrombotic complications of acute infectious diseases, but also is a key trigger for non-infectious cardiovascular diseases. Generally, inflammation triggers the activation of platelets, leukocytes, and endothelial cells (ECs), which can lead to injury or dysfunction of the ECs. Damaged endothelial cells are activated and express various endothelial adhesion molecules, such as ICAM-1 and VCAM-1, which facilitate the attachment of monocytes to HUVECs (Eriksson et al., 2005). In this study, OP has been shown to suppress monocyte adherence to HUVECs (Figure 4A). In accordance with the decreased monocytes adhesion to HUVECs, we noted that OP decreased the protein level of ICAM-1 induced by LPS in HUVECs (Figure 4B). Additionally, inflammation can trigger coagulation pathways, leading to an increased risk of thrombosis. In our research, the observation that OP diminished the retraction of platelet clots triggered by thrombin *in vitro* (Figure 4C) highlighted another significant anti-thrombotic mechanism associated with OP. These findings indicate that OP lowers the level of adhesion molecules in ECs by suppressing the inflammatory response, highlighting its potential to mitigate thrombosis.

TLR4 serves as an innate immune receptor that is involved in the thrombotic process. Existing research has shown that an innate inflammatory cascade response reliant on TLR4 is responsible for triggering thrombosis (Hitchcock et al., 2015). Mice with a targeted deficiency of platelet TLR4 extended intervals to the initial thrombosis and total occlusion (Prakash et al., 2015). This suggests that TLR4 is a promising cross-therapeutic target for anti-inflammatory and antithrombotic mechanisms. Inflammatory responses mediated by TLR4 are strongly associated with the NF-κB signaling pathway. NF-κB is crucial in the inflammatory processes associated with deep vein thrombosis, as it disturbs the homeostasis of ECs and inflammatory responses through the promotion of platelet-inflammatory interactions (Gan et al., 2020). When TLR4 binds to ligand, the NF-κB signaling pathway will be activated and serves as a connector for various inflammatory responses, resulting in the upregulation of proinflammatory factors (Sherif and Al-Shaalan, 2018; Xu et al., 2014). The inflammatory reaction also prompts vascular cells to release adhesion molecules like VCAM-1 and ICAM-1, which subsequently enhances the process of thrombosis (Huang et al., 2024; Eriksson et al., 2005). In terms of cell signaling pathway regulation, known studies have found that OP can control diverse disease progression by regulating FXR/STAT3, MAPK, AMPK, NF-κB and their downstream signaling pathways in multiple targets protein (He et al., 2024; Liao et al., 2019a; Yan et al., 2020c). Previous studies have shown that OP regulates NF-κB and MAPKs signaling pathways by targeting TLR 2/4, thereby activating macrophages to enhance the innate immune response (Chen et al., 2020). In the present investigation, we found that OP effectively suppressed the activation of TLR4/NF-κB and reduced the levels of inflammatory markers such as TNF-α, IL-1β and IL-6 in liver and lung tissues of mice with carrageenan-induced thrombosis. This inhibition partially contributed to the reduction in thrombosis (Figure 5).

Oxidative stress is a significant contributor to vascular endothelial damage, which enhances the adhesion of monocytes to these cells and aids in the formation of blood clots (Nwose et al., 2009; Chen et al., 2009). Extensive study has highlighted the importance of keeping ROS levels low for the homeostasis of healthy blood vessels. Conversely, elevated levels of ROS disturb the balance of oxidative processes in blood vessel cells, which aids in the formation of thrombosis (Bettiol et al., 2022; Gutmann et al., 2020). Studies have shown that antioxidant enzymes such as GPx, CAT, and SOD can relieve vascular oxidative stress caused by ROS, which is a major cause of thrombosis (Loscalzo, 2003; Li et al., 2022; Olsvik et al., 2005). In previous studies, OP has also been found to have significant antioxidant and anti-fatigue effects (Olawuyi and Lee, 2021; Panighel et al., 2022). For instance, OP and xyloglucan hydrogels have been found to have hemostatic and anti-inflammatory effects by scavenging DPPH free radicals (Liu et al., 2024). Meanwhile, OP has been found to inhibit oxidative stress in type 2 diabetes, hypercholesterolemia, and Alzheimer’s disease (Liao et al., 2019a; Panighel et al., 2022; Yan et al., 2020d). Our findings demonstrated that OP enhanced the antioxidant capacity by increasing the content of GSH and upregulating CAT and SOD1/2 expression in the liver and lung tissues of mice induced by carrageenan (Figure 6), which was partly contributed to inhibit thrombosis.

Despite we discovered several intriguing results in this research, there remain certain deficiencies and constraints that need to be addressed. These limitations are as follows: (1) Only a carrageenan-induced mice thrombosis experiment was conducted by us, and it is better to validate the antithrombotic properties of OP in other thrombotic models in future studies, such as those triggered by ferric chloride in the carotid artery and mesenteric vessels; (2) We have only conducted preventive experiments, and the therapeutic effects of OP on antithrombosis requires further investigation; (3) We did not elucidate the pharmacological targets of OP, but we predicted it by using the website of SwissTargetPrediction and found that VEGFA may be a pharmacological target for OP. Hence, it is essential for us to further explore these aspects to enhance and broaden the potential use of OP in the treatment of antithrombosis.

## 5 Conclusion

In summary, we demonstrate that OP, as a natural active compound, is capable of inhibiting thrombosis induced by carrageenan in mice in this study. On one side, OP decreases the expression of ICAM-1 to prevent adhesion of monocytes to damaged ECs and inhibit the retraction of thrombin-induced platelet clots *in vitro*. On the other hand, OP decreased carrageenan-induced inflammation by inhibiting TLR4/NF-κB signaling pathway and enhanced the antioxidant capacity via upregulating SOD1/2 and CAT expression in the liver and lung tissues of mice. These findings not only deepen our understanding of the mechanisms underlying OP’s antithrombotic effects but also highlight its potential clinical relevance and translational significance. Consequently, our research indicates that OP could serve as a promising component in the development of functional foods or therapeutic agents aimed at preventing thrombosis, thereby contributing to the advancement of medical interventions for this condition.

## Data Availability

The original contributions presented in the study are included in the article/Supplementary Material, further inquiries can be directed to the corresponding authors.
