# Supplementary figures and images for: Okra polysaccharide mitigates carrageenan-induced thrombosis in mice by regulating inflammation and oxidative stress
Source: Front Pharmacol. 2025 Apr 10;16:1576108. doi: 10.3389/fphar.2025.1576108 (PMC12018850; doi:10.3389/fphar.2025.1576108)

Figure 3

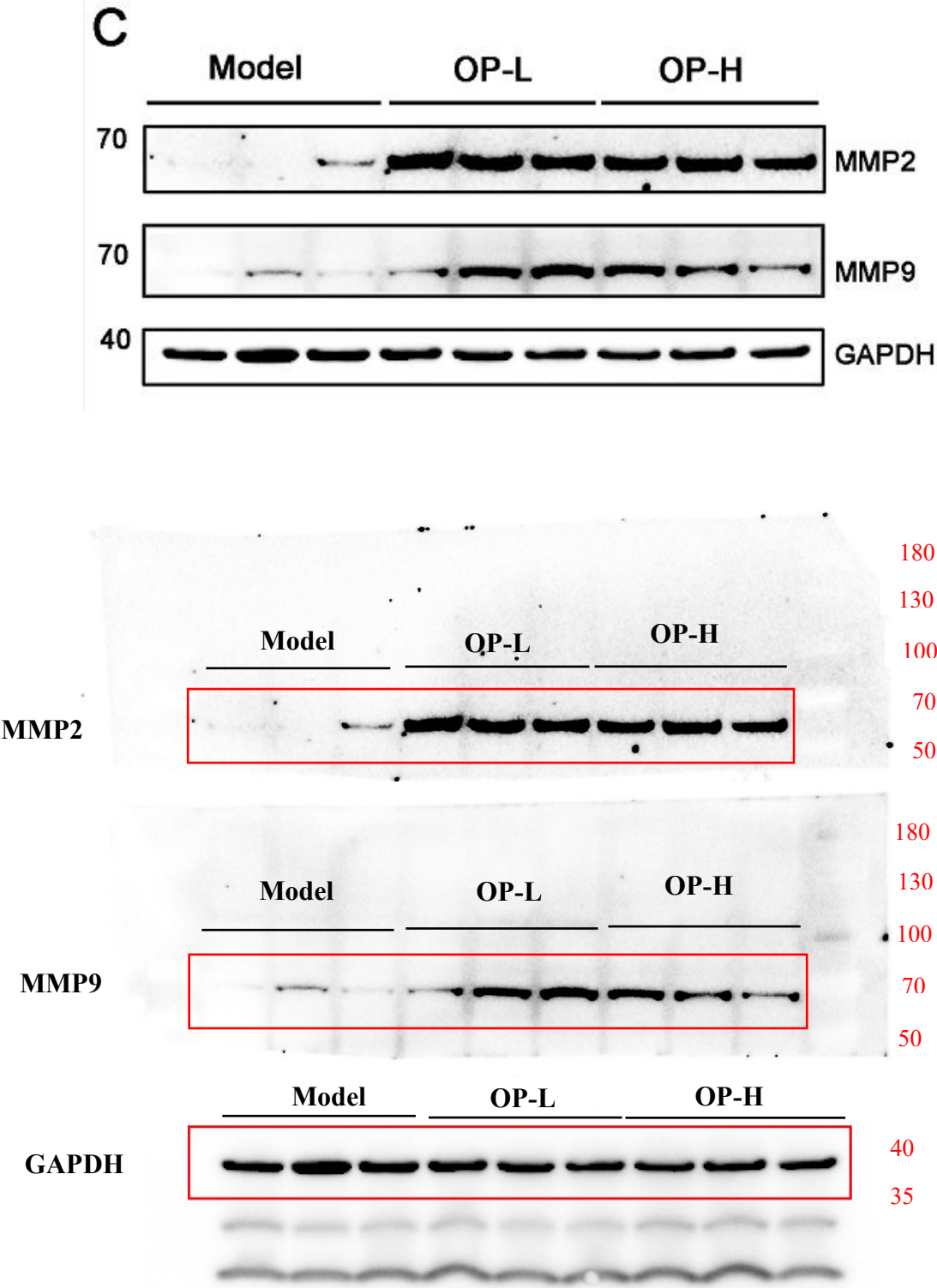

Figure 3

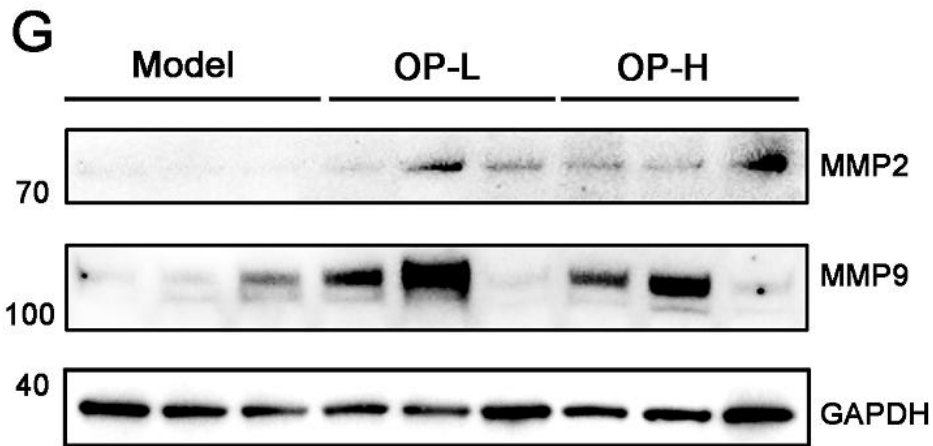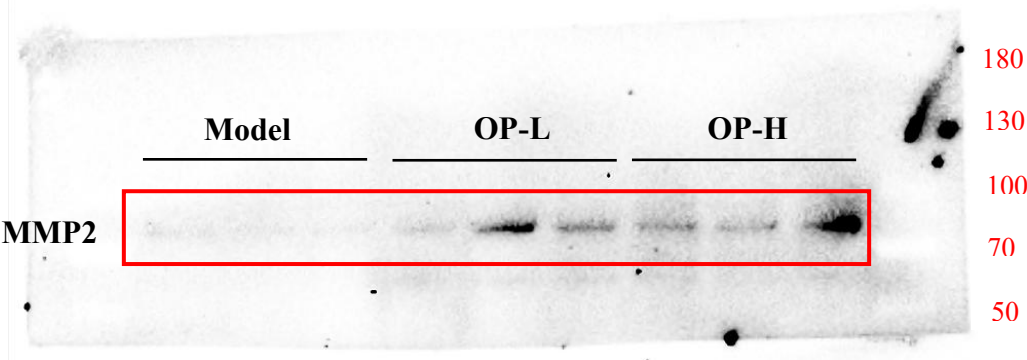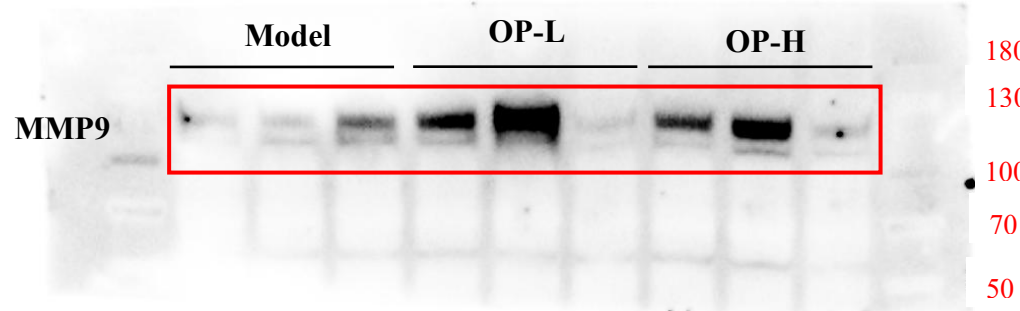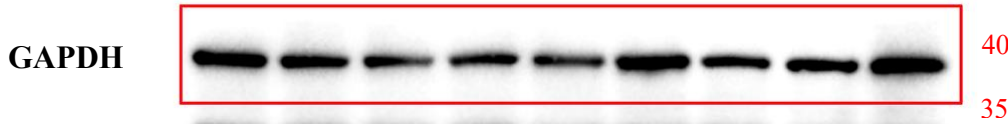

Figure 4

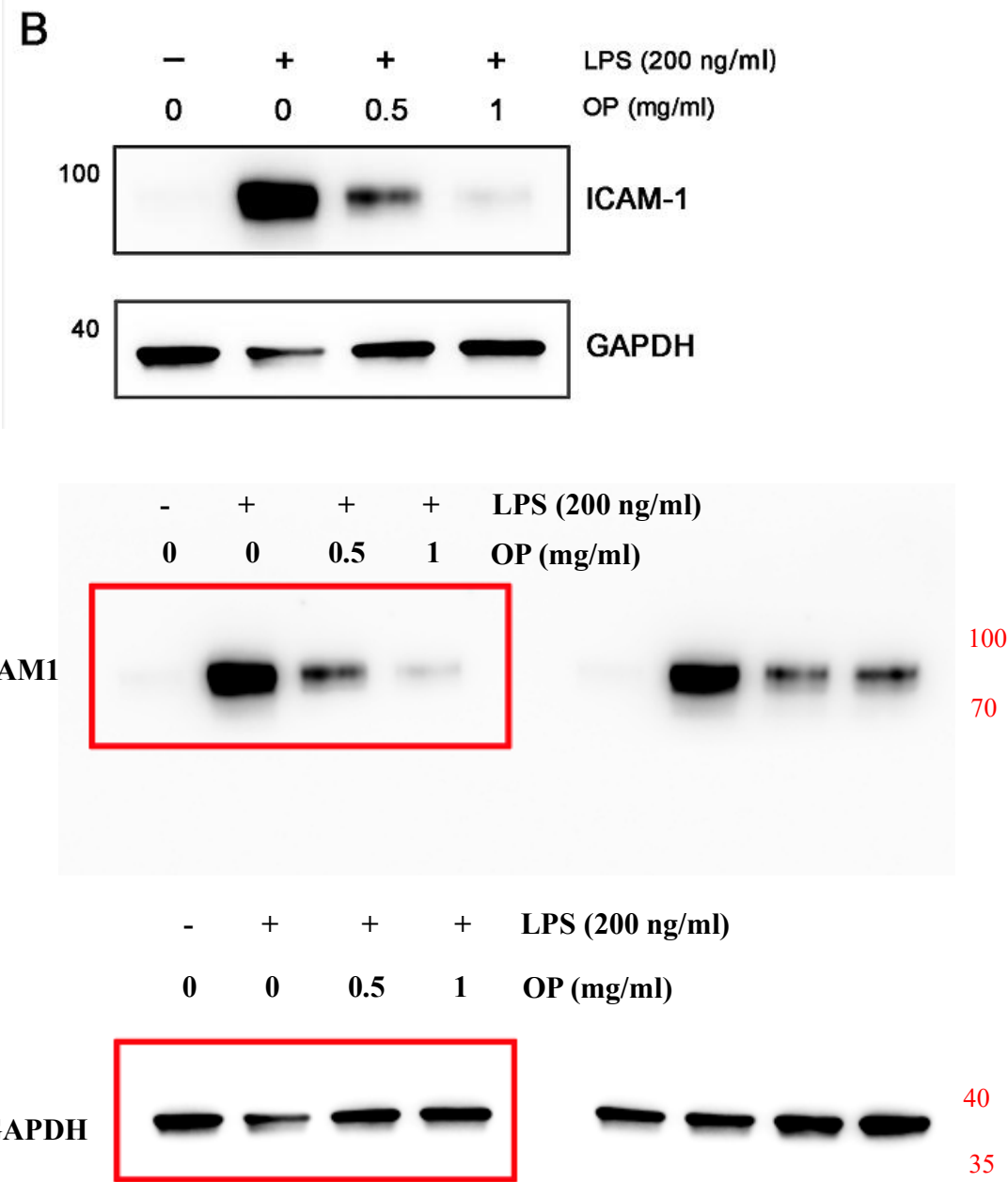

Figure 5

C

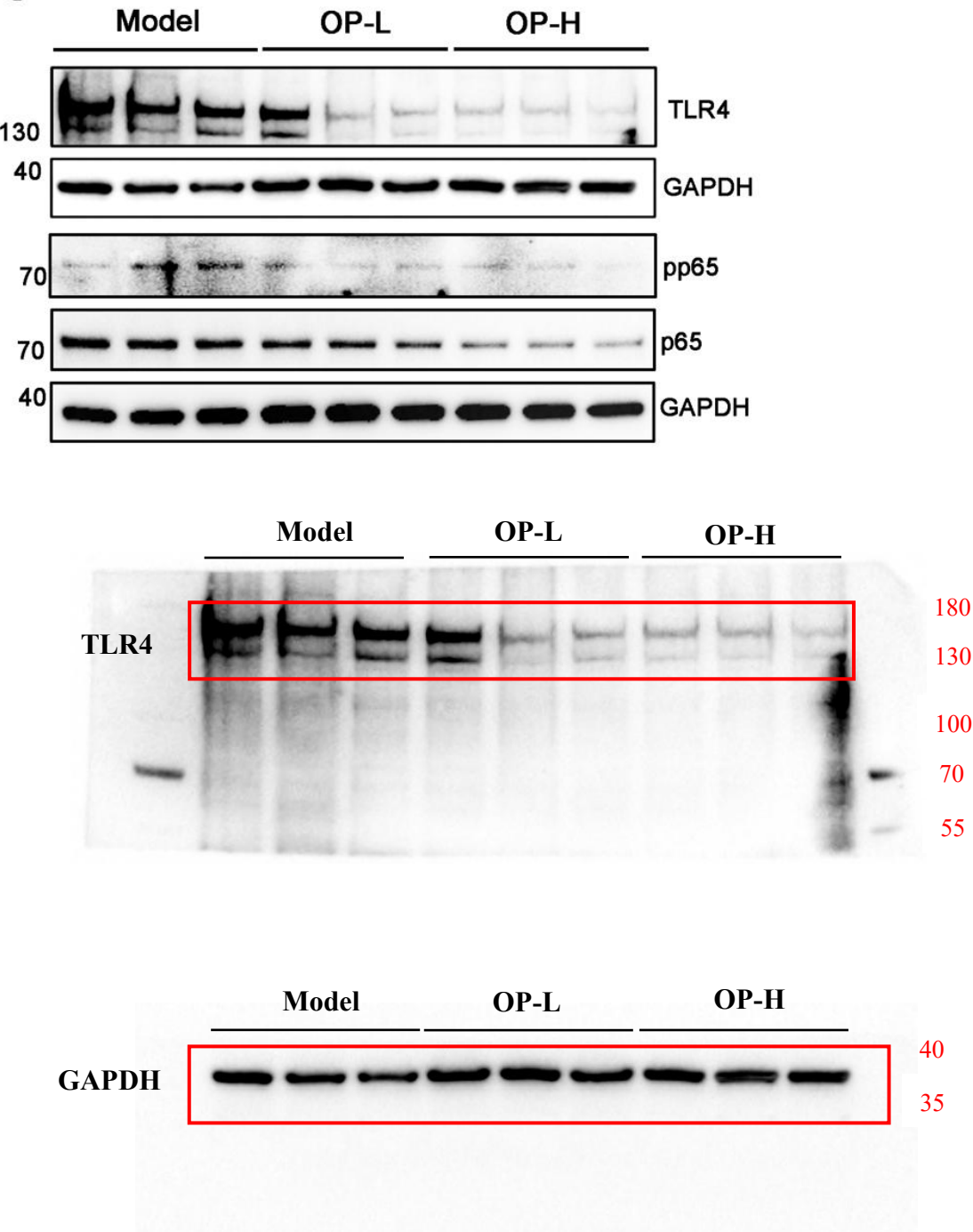

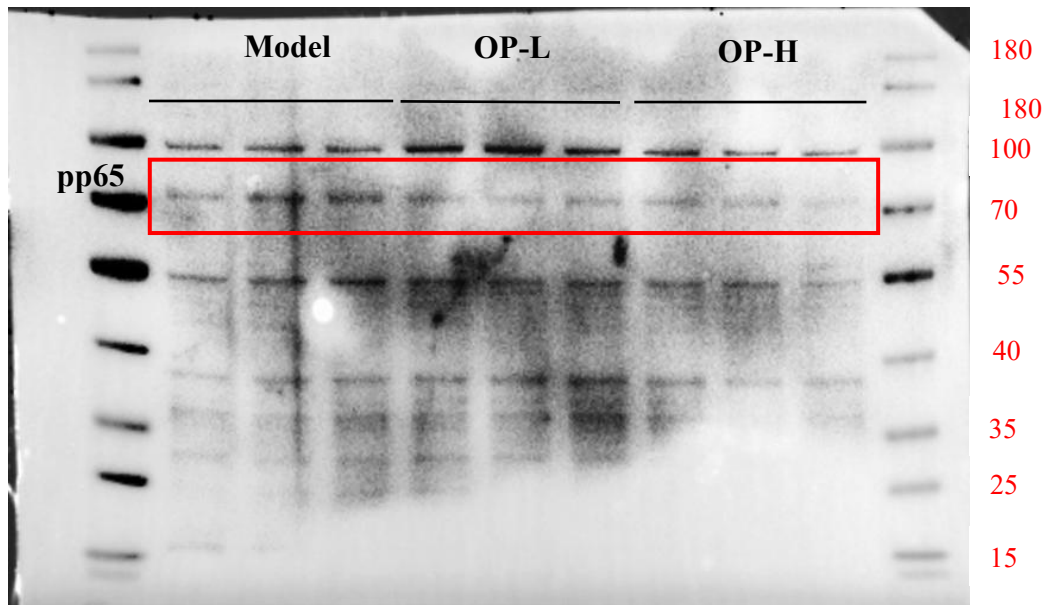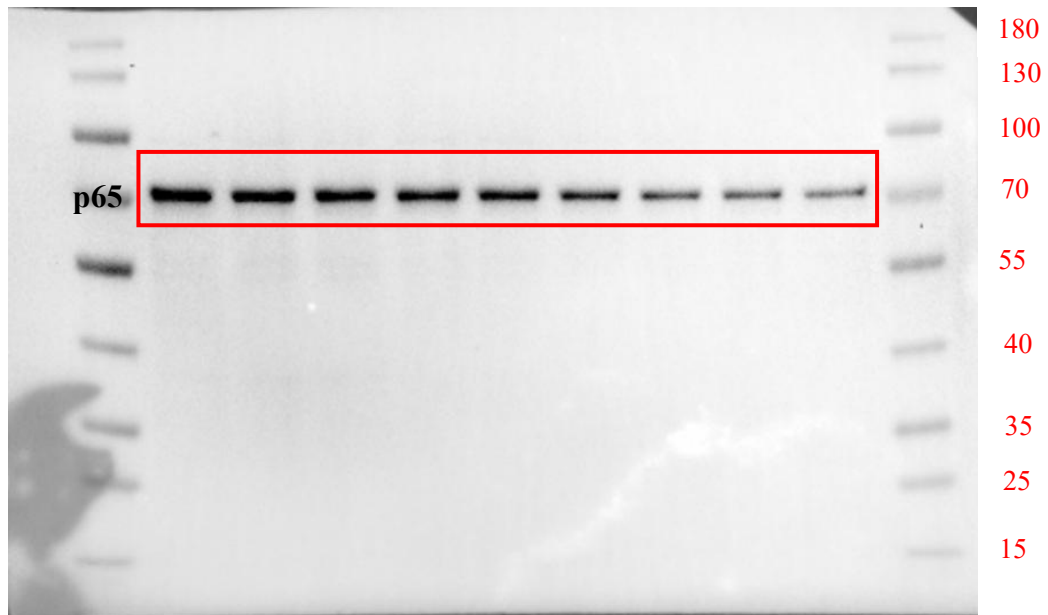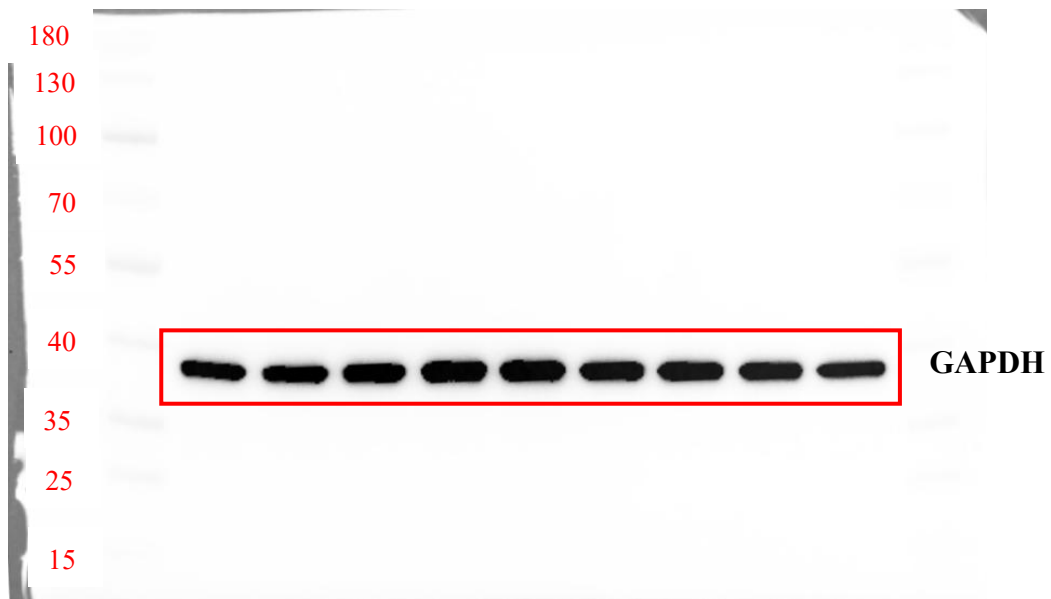

Figure 5

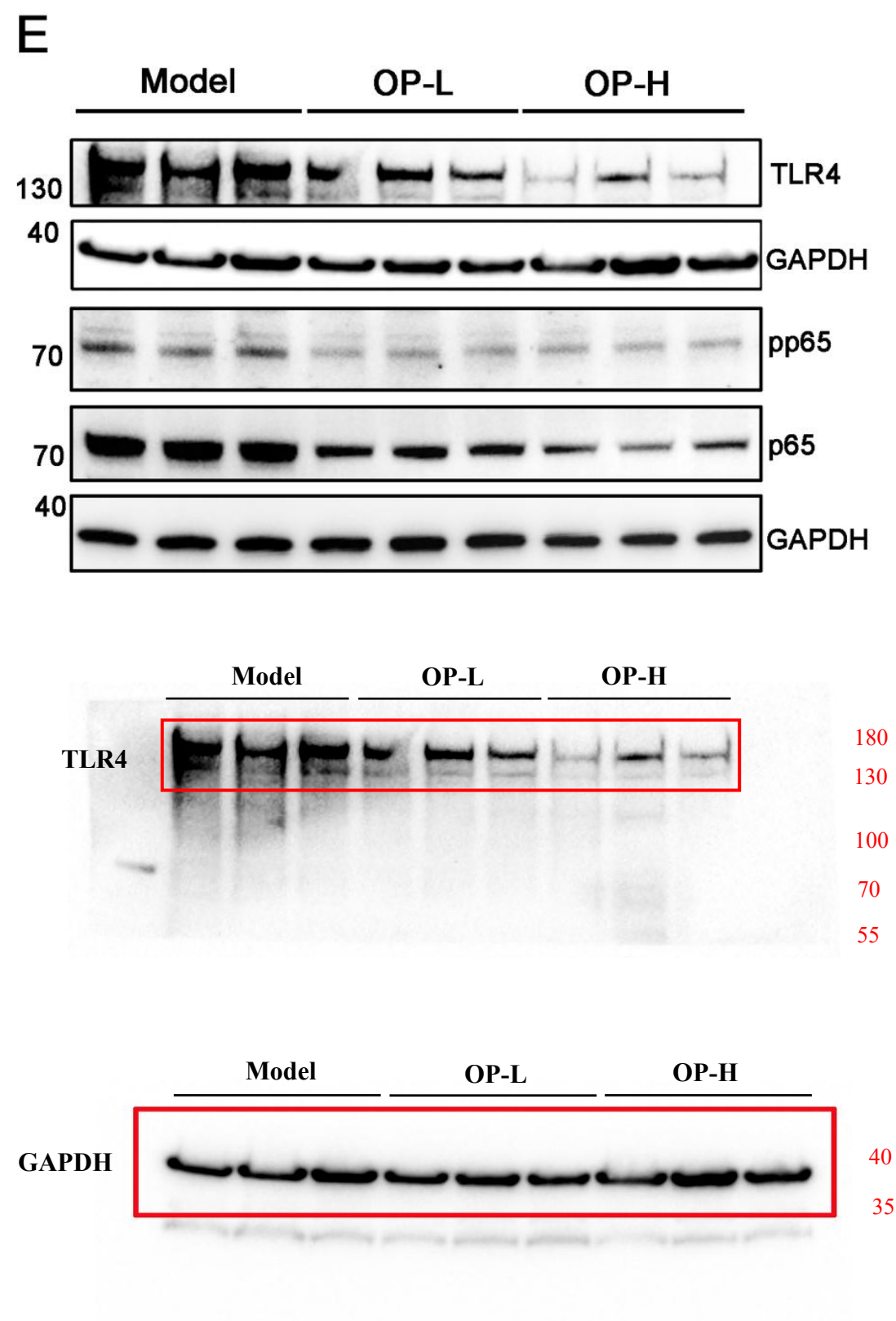

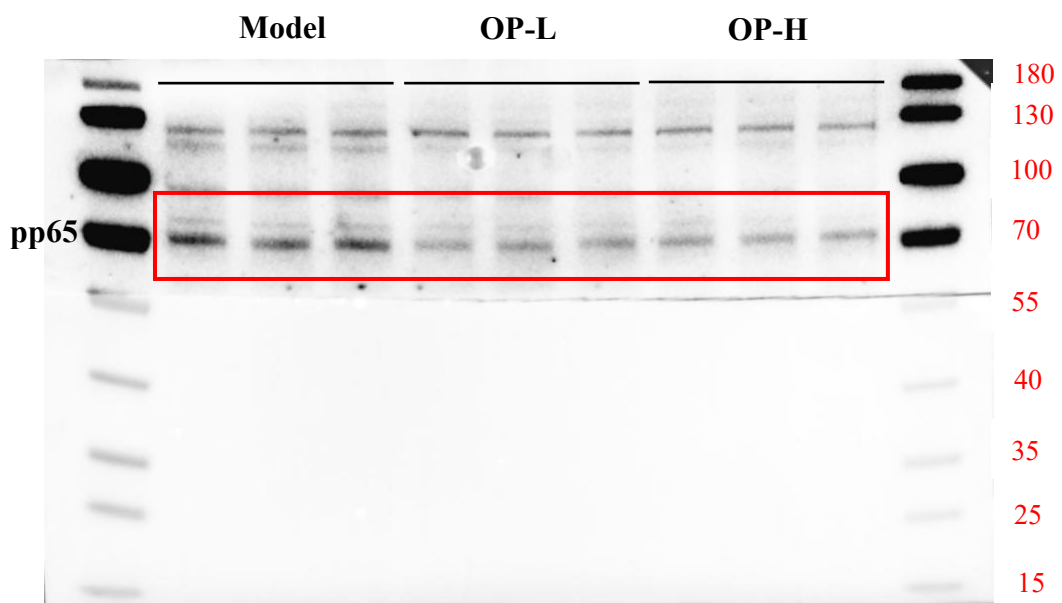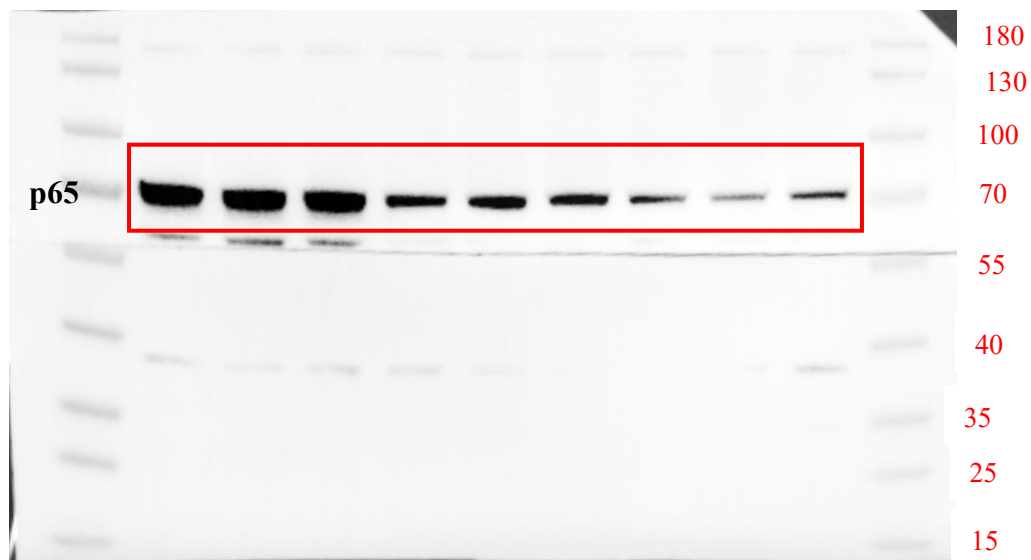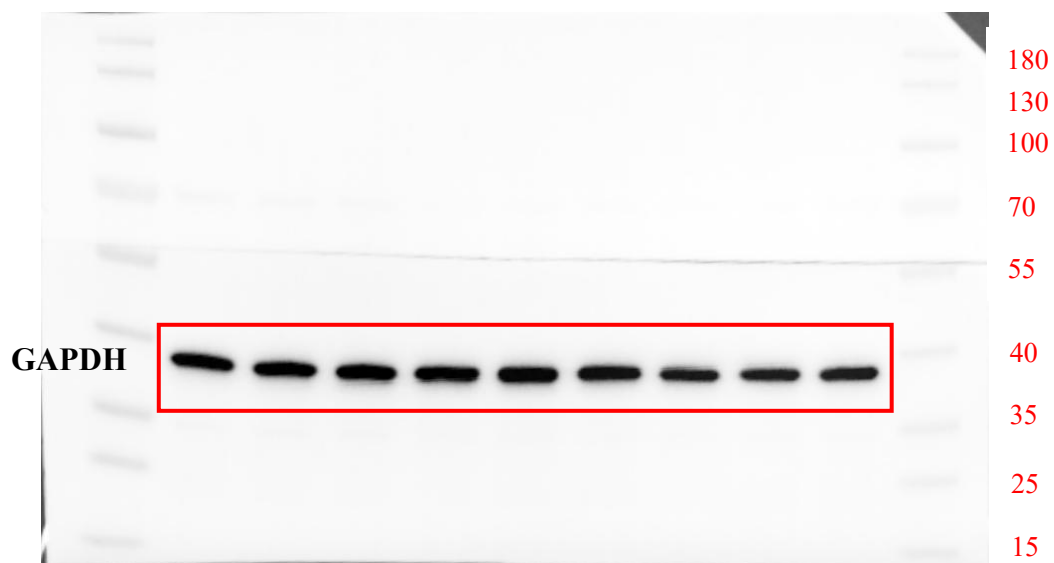

Figure 6

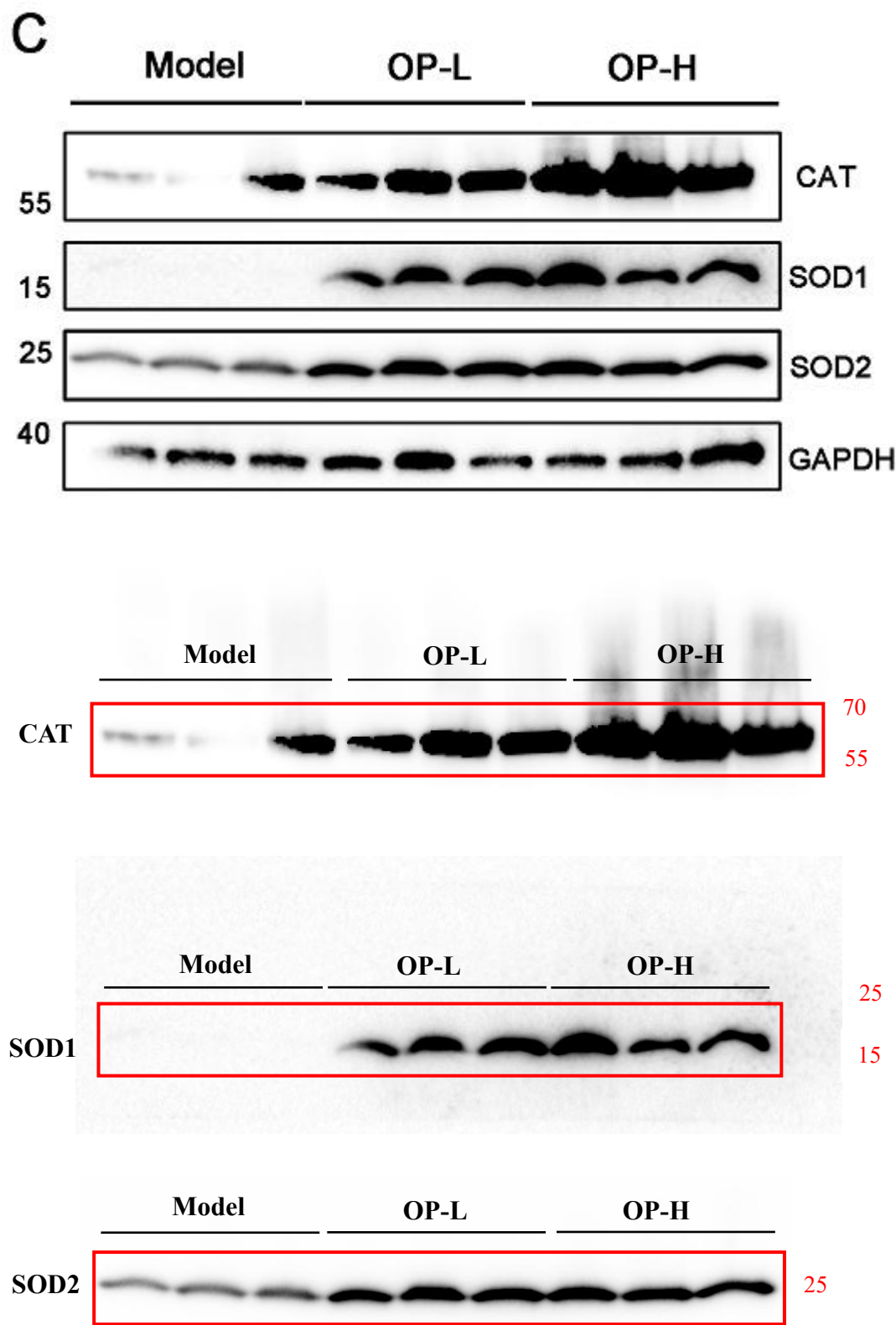

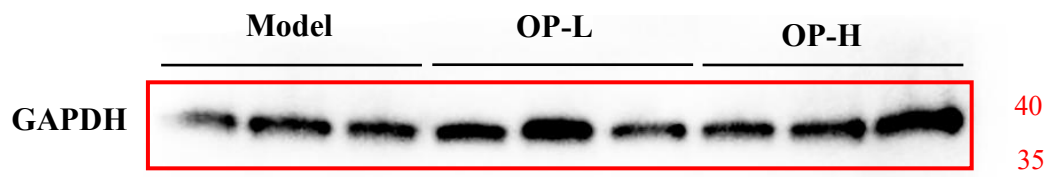

**Figure 6**

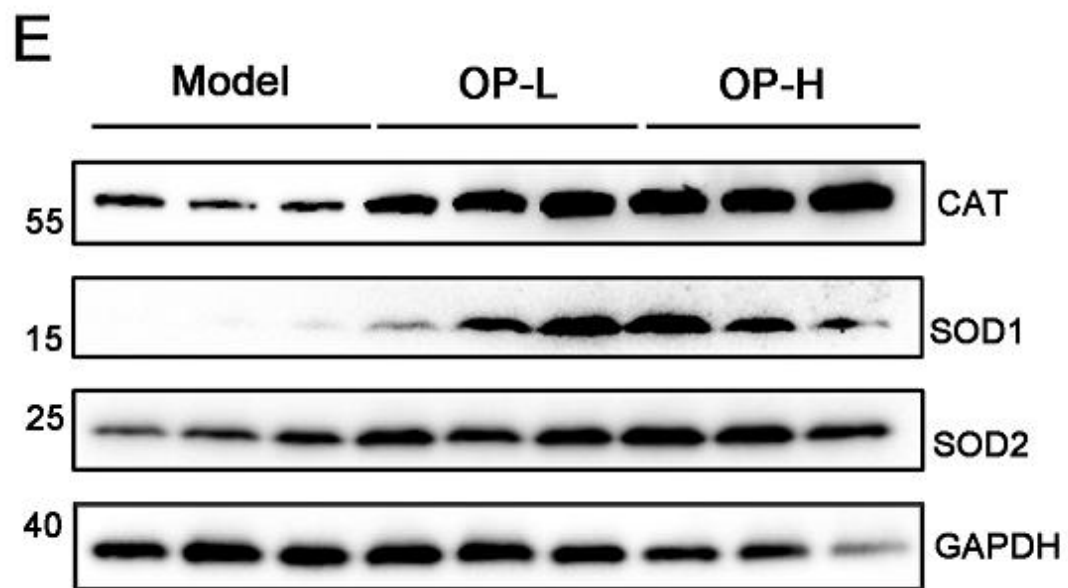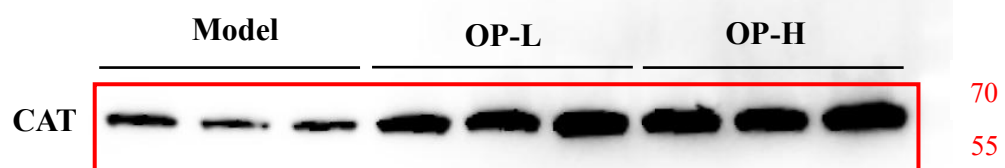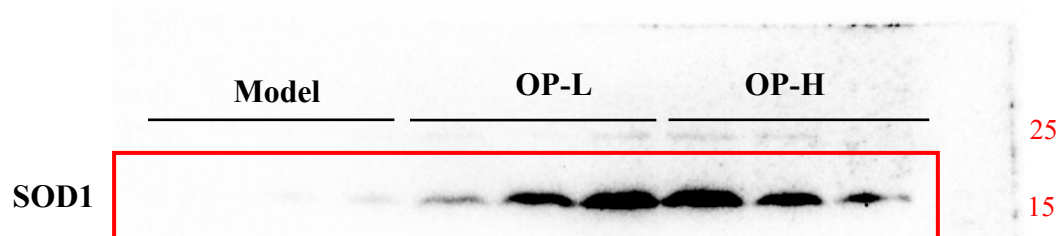

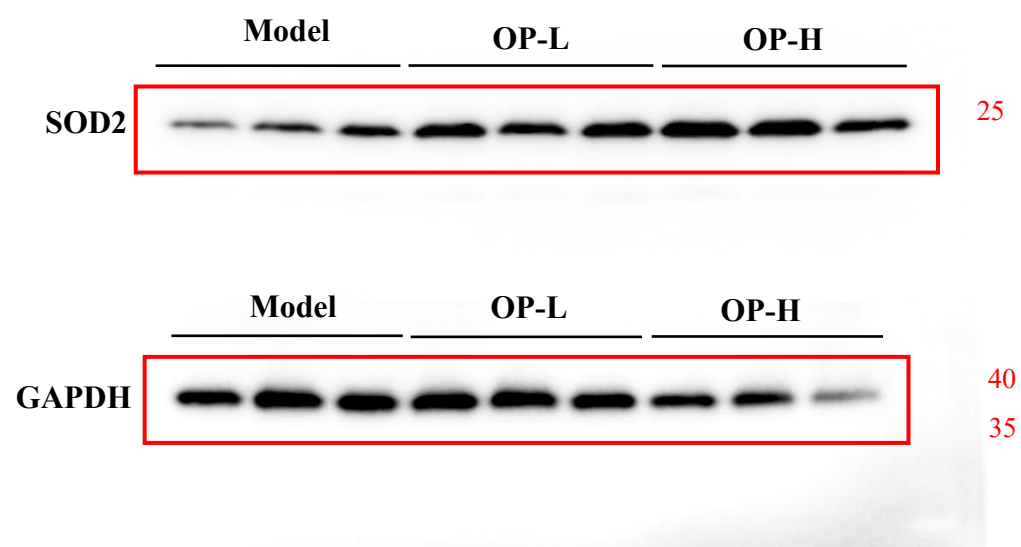

Supplement: Supplementary file 1 [file DataSheet1.pdf]
